# Comparison between catheter ablation versus permanent pacemaker implantation as an initial treatment for tachycardia–bradycardia syndrome patients: a prospective, randomized trial

**DOI:** 10.1186/s12872-024-03920-0

**Published:** 2024-05-10

**Authors:** Min Soo Cho, Ji Hyun Lee, Gi-Byoung Nam, Ki Won Hwang, Myung-Jin Cha, Jun Kim, Kee-Joon Choi

**Affiliations:** 1 Department of Cardiology, Heart Institute, Asan Medical Center, University of Ulsan College of Medicine, 88, Olympic-ro 43-gil, Songpa-gu, Seoul, 05505 Korea; 2 Cardiovascular Center, Seoul National University Bundang Hospital, Seoul National University College of Medicine, Seongnam, Korea; 3 Division of Cardiology, Department of Internal Medicine, Pusan National University Yangsan Hospital, Pusan National University of Medicine, Yangsan, Korea

**Keywords:** Atrial fibrillation, Catheter ablation, Artificial pacemaker, Sick sinus syndrome

## Abstract

**Background:**

Clinical outcomes after catheter ablation (CA) or pacemaker (PM) implantation for the tachycardia–bradycardia syndrome (TBS) has not been evaluated adequately. We tried to compare the efficacy and safety outcomes of CA and PM implantation as an initial treatment option for TBS in paroxysmal atrial fibrillation (AF) patients.

**Methods:**

Sixty-eight patients with paroxysmal AF and TBS (mean 63.7 years, 63.2% male) were randomized, and received CA (*n* = 35) or PM (*n* = 33) as initial treatments. The primary outcomes were unexpected emergency room visits or hospitalizations attributed to cardiovascular causes.

**Results:**

In the intention-to-treatment analysis, the rates of primary outcomes were not significantly different between the two groups at the 2-year follow-up (19.8% vs. 25.9%; hazard ratio (HR) 0.73, 95% confidence interval (CI) 0.25–2.20, *P* = 0.584), irrespective of whether the results were adjusted for age (HR 1.12, 95% CI 0.34–3.64, *P* = 0.852). The 2-year rate of recurrent AF was significantly lower in the CA group compared to the PM group (33.9% vs. 56.8%, *P* = 0.038). Four patients (11.4%) in the CA group finally received PMs after CA owing to recurrent syncope episodes. The rate of major or minor procedure related complications was not significantly different between the two groups.

**Conclusion:**

CA had a similar efficacy and safety profile with that of PM and a higher sinus rhythm maintenance rate. CA could be considered as a preferable initial treatment option over PM implantation in patients with paroxysmal AF and TBS.

**Trial registration:**

KCT0000155.

## Background

The tachycardia–bradycardia syndrome (TBS) is a variant of sick sinus syndrome characterized by alternating tachycardia and bradycardias [1]. Specifically, the long pause after the termination of the episode of tachyarrhythmia is the characteristic feature of TBS and is usually associated with symptoms such as dizziness or syncope. Atrial fibrillation (AF), the most common tachyarrhythmia in patients with TBS, results from electrophysiological and structural remodeling of atrium, which triggers both AF and sinus node dysfunction [2, 3]. In view of the aging of the general population, its importance has become significant. The treatment of TBS usually requires the implantation of a permanent pacemaker (PM) as medications targeting tachyarrhythmia worsen the underlying sinus node dysfunction (SND) [4, 5]. However, PM implantation is also associated with procedure-related complications, such as vascular damage, pneumothorax, lead dislodgement, or perforation. In addition, there is an ongoing risk of long-term device-related complications, such as lead or pocket infections, pacemaker syndrome, left ventricular (LV) dysfunction, and tricuspid valve damage [6].

Recently, as an alternative to the conventional PM-based treatment, a catheter ablation (CA)-based approach emerged as a new approach in the management of the TBS [7–9]. The CA incorporating pulmonary vein isolation (PVI) has been known to reduce the symptoms from TBS and obviates the need for PM implantation [10, 11]. Recent observational data comparing CA and PM as an initial treatment for TBS suggested that CA was related to the higher rate of sinus rhythm maintenance with similar cardiovascular outcomes to the PM-based treatment [12, 13]. However, most of the previous studies were associated with an inherent selection bias wherein the CA procedure was usually performed in younger patients with lower comorbidities. Therefore, to verify whether CA could be a preferable initial treatment over PM implantation in patients with TBS, data from randomized controlled trials are required. Accordingly, we planned a prospective randomized controlled pilot study to compare the efficacy and safety outcomes after CA and PM implantation as initial treatments for TBS associated with AF.

## Methods

This was a prospective, single-center, open-label, randomized controlled trial study, conducted to evaluate the efficacy and safety of CA compared to the PM implantation. The study protocol was approved by the institutional review board of Asan Medical Center, Seoul, Korea (2010 − 0768). Written informed consent was provided by all participants. This study conformed to the ethical guidelines of the Declaration of Helsinki 2013. The trial has been registered on the International Clinical Trials Registry Platform of South Korea (cris.nih.go.kr, Registration Number: KCT0000155, Date of Registration: 28/07/2011).

The current study included patients with ages ≥ 40 years, documented episodes of both paroxysmal AF and post-tachycardia pauses > 3 s, and presence of symptoms related to the pause. Both AF and post-tachycardia pauses should be documented on any forms of electrocardiography (ECG), such as 12-lead ECG, Holter monitoring, or telemonitoring during hospitalization. The exclusion criteria for the study included left ventricular ejection fractions < 40%, prior CA procedures for AF, presence of LA thrombi, congestive heart failure (New York Heart Association classes III or IV), revascularization for the coronary artery disease within 6 months, contraindications to anticoagulation, pregnancy, and a life expectancy < 12 months.

We randomly assigned patients with paroxysmal AF in a 1:1 ratio to open-label treatments with either CA or PM using a computerized randomization system. The randomization sequence was computer generated with a block size of 4 or 6. After randomization, all patients underwent CA or PM according to the assignment within 3 days. In the CA group a three-dimensional (3D) electroanatomic mapping system (EnSite NavX, Abbott, St. Paul, MN, USA) was used for left atrial mapping and ablation. Radiofrequency (RF) pulses were delivered with a 3.5 mm, open-irrigated tip-ablation catheter (Coolflex, Abbott, St. Paul, MN, USA). RF powers from 30 to 35 W were used for ablation of anterior pulmonary vein (PV) antrum, carina, and ridge, and powers equal to 25 W were used for the posterior wall or near the esophagus. Circumferential PV isolation was mandatory for all patients and was performed 5–10 mm outside the PV ostia. The endpoint of the PV isolation was the elimination or dissociation of the PV potentials. The extents or types of extra-PV ablation lesions were decided upon the physician’s discretion. All patients were monitored using 24 h Holter monitoring on the day after the procedures. Patients were seen in the outpatient clinic at 1, 3 and 6 months after the procedures, and every 6 months thereafter. Standard 12-lead ECGs and Holter monitoring were conducted at every outpatient visit, and additional recordings were conducted if needed, depending on the patient’s symptoms. Antiarrhythmic drugs were discontinued after the procedures to assess the risk of recurrent syncope of the study population but could be used in limited cases upon the attending physician’s discretion. Anticoagulants were administered at the discretion of the attending physician.

In the PM group, dual-chamber pacemakers were principally implanted and atrial lead was implanted at the right atrial appendage and RV lead at the right ventricular apex using a standard technique [14]. The patients were programmed to the DDD mode at the time of discharge. The patients were followed in the outpatient clinic based on the same follow-up ECG and Holter monitoring schedule as that which related to the CA group. Interrogation data of the PM were acquired at every visit, including the data which related to recurrent tachyarrhythmia detected by the PM device. The patients in the PM group could use the antiarrhythmic drugs freely according to the patient symptoms and recommended to maintain the sinus rhythm. The methods for the anticoagulation and follow-up therapies were the same as those adopted for the CA group.

The primary end point of the study was unexpected emergency room (ER) visit or re-hospitalization from any cardiovascular causes. The secondary endpoint included all-cause mortality, rate of recurrent atrial fibrillation or tachycardia (AF/AT), and the rate of procedure-related complications. The AF/AT recurrence was diagnosed when a sustained episode which lasted > 30 s was documented on the standard electrocardiogram or Holter monitor, either routine or symptom driven, after a blanking period of 3 months in both groups [7]. The episodes of subclinical AF in the PM group (based upon the suggested criteria of manufacturers), which lasted more than 6 min, were analyzed separately as a secondary clinical endpoint [15].

This study was a pilot study conducted to evaluate the feasibility of CA as an alternative to the PM in patients with TBS. We aimed to enroll 70 patients, 35 in each strategy according to consensus reached by researchers. Primary analysis was performed on an intention-to-treat basis. Adverse events were assessed in all randomized patients after the index procedure. Categorical variables are presented as frequencies with percentage, and continuous variables as median and interquartile ranges, or as means and standard deviations. Comparisons between groups were conducted using the Chi-square or Fisher’s exact tests for categorical variables, and the Student’s t-test for continuous variables. All statistical analyses were performed using the software R (version 3.3.1, R foundation, Vienna, Austria) and two-sided P-values < 0.05 were considered statistically significant.

## Results

**Fig. 1:**
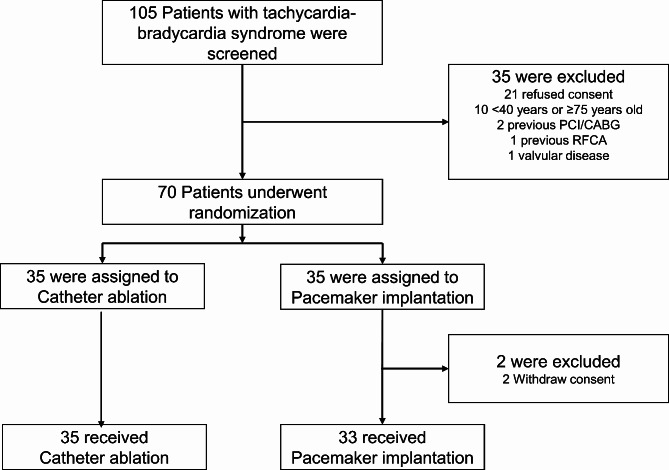
Study flow diagram. Of the 105 patients with tachycardia-bradycardia syndrome, 35 were excluded from the study for the following reasons; 21 refused consent, 10 < 40 years or ≥ 75 years, 2 previous percutaneous coronary intervention or coronary artery bypass grafting, 1 previous catheter ablation, and 1 valvular heart disease. Of the 70 patients who underwent randomization, 35 patients were assigned to catheter ablation and 35 to the pacemaker group. Two patients in the pacemaker group withdrew consent immediately after the randomization and asked for their data to be deleted. The remaining 68 patients were included in the intention-to-treat analysis

The numbers of patients who were screened, randomized, and assigned to each study group are shown in Fig. 1. Between March 2013 and December 2016, we enrolled 70 patients, who were then randomly assigned to either the CA group (*n* = 35) or the PM group (*n* = 35). After the exclusion of two patients who withdrew their consents immediately after the randomization, a total of 68 patients were left for primary analysis.

The baseline characteristics of the participants are summarized in Table 1. The mean age of overall population was 63.4 ± 7.1 years old and 63.2% were male. All patients had a history of paroxysmal AF and the median CHA_2_DS_2_-VASc score was equal to 2 (interquartile range [IQR] 1–3). Half of the patients (51.5%) had experienced episodes of syncope, and the maximal pause after AF documented on Holter or electrocardiography monitoring was 5.0 ± 2.0 s. In the between group comparison, the ages of patients in the CA group were slightly but significantly younger than those of the PM group participants (61.5 ± 7.6 vs. 65.4 ± 6.6 years, *P* = 0.020). All other characteristics were well balanced between the two groups.

**Table 1:** Baseline characteristics of studied patients

	Catheter ablation (*n* = 35)	Permanent pacemaker (*n* = 33)	*P* value
Age	61.5 ± 7.6	65.4 ± 6.6	0.020
Male	24 (68.6)	19 (57.6)	0.347
Body mass index	24.7 ± 2.9	25.1 ± 3.9	0.587
Past medical history			
Congestive heart failure	0 (0.0)	1 (3.0)	0.299
Hypertension	19 (54.3)	18 (54.5)	0.983
Diabetes	5 (14.3)	7 (21.2)	0.454
Stroke	2 (5.7)	5 (15.2)	0.201
Vascular disease	3 (8.6)	5 (15.2)	0.400
Syncope	16 (45.7)	19 (57.6)	0.328
Maximal pause, sec	4.8 ± 1.9	5.2 ± 2.1	0.446
Hemoglobin level, mg/dL	14.0 ± 1.7	13.7 ± 1.8	0.575
Left atrial anterior-posterior diameter, mm	40.6 ± 3.8	41.1 ± 5.6	0.653
Left ventricular ejection fraction, %	60.3 ± 7.2	62.0 ± 7.5	0.365
Tricuspid regurgitation			0.862
No	23 (65.7)	22 (66.7)	
Mild	10 (28.6)	10 (30.3)	
Moderate	2 (5.7)	1 (3.0)	
E/E’	10.5 ± 3.7	11.6 ± 5.0	0.340

All patients in both groups received the initially assigned treatments, and no crossover occurred. In the CA group, PV isolation was successful in all patients, and 26 patients underwent CTI ablation. Additional linear, CFAE, or trigger ablations were performed in 15, 1, and 5 patients, respectively. In the PM group, all patients received dual-chamber pacemakers. Upon discharge, antiarrhythmic medications were more frequently used in the PM group compared with the CA group (75.8% vs. 22.9%, *P* < 0.001).

**Fig. 2:**
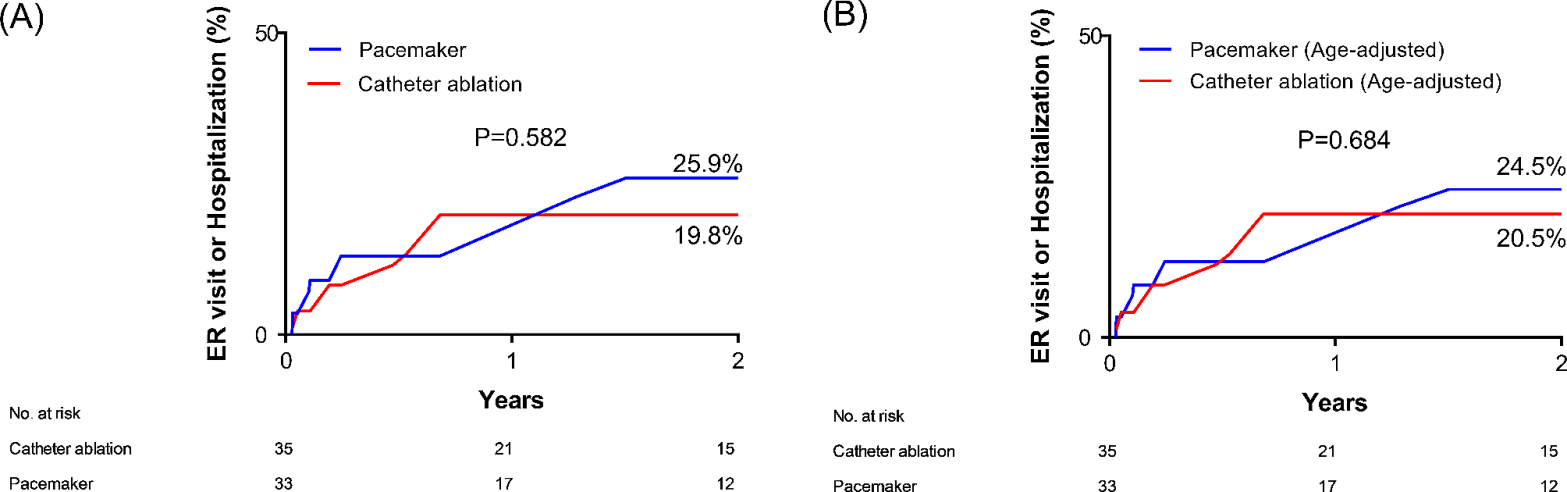
(**A**) Rate of unexpected emergency room visits or rehospitalizations, and (**B**) age-adjusted rate of unexpected emergency room visits or rehospitalizations

In the analysis of primary endpoints of unexpected ER visits or hospitalization, there were no significant differences between the CA and PM groups during the 2-year follow-up (19.8% vs. 25.9%, respectively, hazard ratio [HR] 0.73, 95% confidence interval [CI] 0.25–2.20, *P* = 0.584, Fig. 2). The risk of the primary endpoint was consistent when the result was adjusted for the age (HR 1.12, 95% CI 0.34–3.64, *P* = 0.852, Fig. 2). The details of the primary endpoint are summarized in Table 2. In the CA group, primary endpoints occurred in eight patients. Four patients experienced syncope and underwent pacemaker implantation. Three other patients visited the hospital for recurrent atrial tachyarrhythmias and one patient for pericarditis. In the PM group, three patients experienced PM related complications, such as pocket infection, lead displacement, and wound hematoma. Another three patients visited the hospital for atrial tachyarrhythmia. The number of patients with unexpected ER visits or hospitalization due to syncope was numerically larger in the CA group (11.4% vs. 0%, *P* = 0.115), whereas the difference due to symptomatic AF/AT was not significant (8.6% vs. 9.1%, *P* > 0.99). One patient in the PM group underwent CA during the study period due to symptomatic AF/AT that was refractory to antiarrhythmic medications. Unexpected hospital visits not related to the procedure occurred in four patients (two stroke or systemic embolism, one chest pain, and one warfarin intoxication patients).

**Table 2:** Details of unexpected emergency room (ER) visits or rehospitalizations

Catheter ablation	*n* = 35
Pacemaker implantation due to syncope	4 (11.4)
Atrial tachyarrhythmia	3 (8.6)
Pericarditis	1 (2.9)

**Permanent pacemaker**	*n* = 33
Pacemaker infection	1 (3.0)
Lead displacement	1 (3.0)
Wound hematoma care	1 (3.0)
Stroke or systemic embolism	2 (6.1)
Angina	1 (3.0)
Atrial tachyarrhythmia	3 (9.1)
Prolonged INR	1 (3.0)

**Fig. 3:**
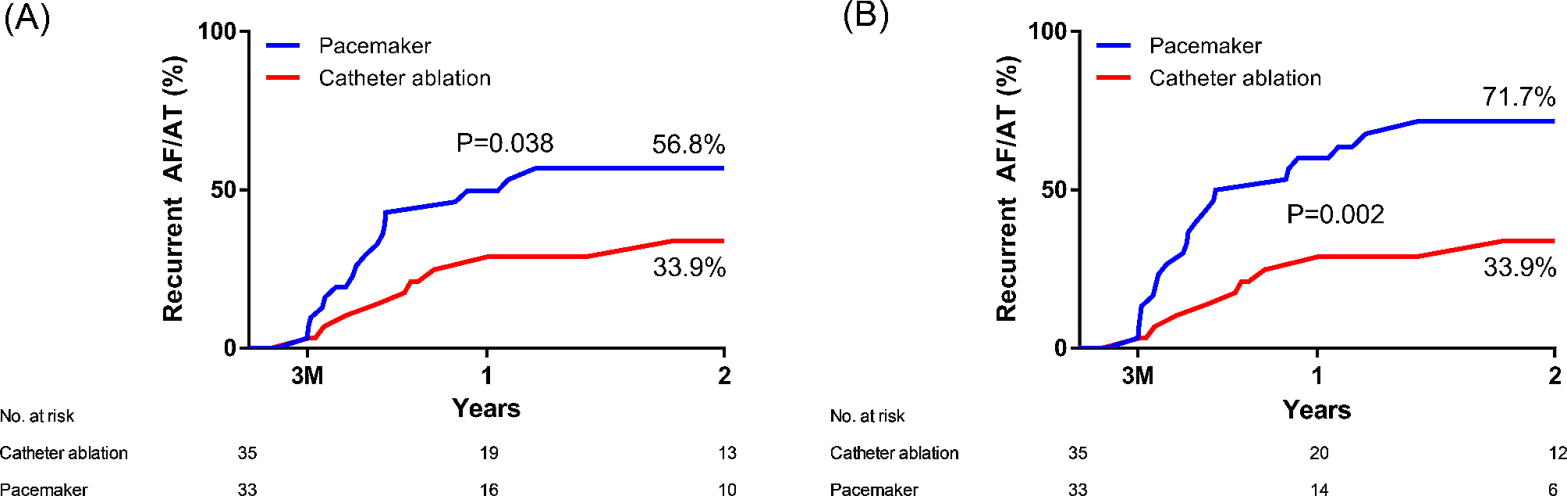
Rate of (**A**) clinical atrial fibrillation or tachycardia recurrence, and (**B**) clinical or subclinical atrial fibrillation or tachycardia recurrence

The rate of recurrent AF/AT after 3 months of blanking period was significantly higher in the PM group compared with the CA group (33.9% vs. 56.8%, *P* = 0.038, Fig. 3). The higher rate of recurrent AF/AT in the PM group was more exaggerated when the subclinical AFs were regarded as AT/AF recurrences (33.9% vs. 71.7%, *P* = 0.002, Fig. 3). The rates of procedure-related complications were not significantly different between the two groups (14.2% [5/35] vs. 15.2% [5/33], *P* = 0.920). The cardiac tamponade (*n* = 3), pericarditis (*n* = 1), and access site hematoma (*n* = 1) occurred in the CA group, and wound hematoma (*n* = 3), lead dislodgment (*n* = 1), and device-related infection (*n* = 1) in the PM group. All patients with cardiac tamponades were successfully resuscitated with pericardiocentesis. No one died throughout the study period.

## Discussion

In this open-label, randomized trial, the rates of unexpected ER visits or hospitalizations were similar after CA or PM treatments in patients with paroxysmal AF with symptomatic long pauses. In addition, CA was associated with a higher rate of sinus rhythm maintenance with similar rates of procedure-related complications.

TBS is a clinically unique disease as alternating tachycardia and bradycardia affect each other, and the medications for the tachycardia could worsen the episodes of bradycardia. Patients with AF demonstrate fibrosis in their sinus node and atrial tachycardia itself leads to the downregulation of the hyperpolarization-activated cation channel (HCN 4), which is the predominant subtype in the SAN [16, 17]. Conversely, SND could lead to the atrial tachyarrhythmia as generalized fibrosis of cardiac chamber leads to the various forms of tachyarrhythmia [18–20]. As the atrial tachycardia could be an aggravating factor of SND, the suppression of tachyarrhythmia was attempted in many previous trials, and consistently demonstrated the partial restoration of sinus node function and obviation of PM [10, 11]. Chen et al. reported that the CA could be a superior treatment strategy compared with PM, as it can reduce the tachycardia-related hospitalizations and use of antiarrhythmic medications [12]. A recent study from our group reported long-term outcomes after CA or PM as an initial treatment strategy [13]. During the 3-year follow-up period, there were no differences in rehospitalizations or deaths (20.5% vs. 20.0%, *P* = 0.646), but CA was superior in its capacity to reduce the AF/AT recurrence (64.7% vs. 25.7%, *P* < 0.001). More importantly, the crossover was only 7.4% in the CA arm during the study period, thus suggesting that CA could be an effective alternative for the PM [21]. However, our and other previously published studies had inherent selection biases from observational studies, as CA patients were usually rhythm control-eligible patients at younger ages associated with lower rates of comorbidities. Therefore, a randomized study was required to verify that RFCA could be a preferable treatment option compared with PM-based strategies.

From this regard, our study has considerable clinical value as it directly assessed the value of CA as an initial treatment strategy for patients with TBS from AF. In our study, the CA demonstrated similar rates of unexpected rehospitalizations or ER visits, but it was superior in terms of the sinus rhythm maintenance; furthermore, most of CA arm (88.6%) could avoid PM implantation during the study periods. The complication rates were similar, but the rate associated with the CA arm patients could be reduced more as PVI-only ablations were more frequently used recently after the STAR-AF II trial [22]. Considering that the sinus rhythm maintenance could be associated with better quality of life, exercise tolerance, and with reduced rates of cardiovascular events [23, 24], we believe that the CA could be a preferable treatment option over PM in patients with TBS associated with paroxysmal AF.

The mechanism responsible for the beneficial effects of CA on SND is beyond the scope of the current study but can be partly explained based on several ways. The reduction of the AF event itself could reduce the number of long-pauses after tachycardia termination. Recent AF studies using ILR clearly demonstrated excellent outcomes in terms of the reduction of the AF burden [25]. This reduction of AF burden could have also resulted in reverse-remodeling of sinus node through the negation of the AF related sinus node dysfunction [10]. Another possible mechanism could be attributed to vagal denervation from wide PV ablation. The ganglionic plexuses (GP) were located near the PV in the human heart, and wide PV ablation could have resulted in vagal denervation [26]. Qin et al. already demonstrated that intentional GP ablation effectively increased the sinus rate in patients with significant sinus bradycardia [27]. These mechanistic explanations suggest that CA could reverse the pathophysiologic change of the sinus node, and can ultimately decrease the complications from TBS.

Our study is associated with several limitations. The current study is fundamentally a pilot study for the formulation of a hypothesis and cannot provide sufficient power for supporting a specific strategy. There was subtle difference in the mean age between the two study groups which may be attributed to the small number of study participants. However, we believe that our results are still valid as the absolute differences between the two groups were only a few, other comorbidities and the length of post-tachycardia pause were well balanced, and clinical outcomes were consistent even after adjustments for age. As most parts of the study were conducted before the STAR-AF II trial, a more aggressive ablation strategy was applied, which led to a relatively higher rate of CA-related complications, such as cardiac tamponade.

## Conclusions

CA was associated with a similar efficacy and safety with a higher rate of sinus rhythm maintenance compared with PM implantation in patients with TBS. Therefore, CA could be considered a preferable initial treatment option compared with PM implantation.

## Data Availability

The original contributions presented in the study are included in the article/Supplementary Material, further inquiries can be directed to the corresponding author. The detailed data related to the findings of this study are available from the corresponding author upon reasonable request.
